# Hybrid Histogram Descriptor: A Fusion Feature Representation for Image Retrieval

**DOI:** 10.3390/s18061943

**Published:** 2018-06-15

**Authors:** Qinghe Feng, Qiaohong Hao, Yuqi Chen, Yugen Yi, Ying Wei, Jiangyan Dai

**Affiliations:** 1 College of Information Science and Engineering, Northeastern University, Shenyang 110004, China; 1510377@stu.neu.edu.cn; 2 School of Computer Science and Technology, Tianjin University, Tianjin 300350, China; qiaohonghao@gmail.com; 3 School of Software, Jiangxi Normal University, Nanchang 330022, China; 005090@jxnu.edu.cn (Y.C.); yiyg510@jxnu.edu.cn (Y.Y.); 4 School of Computer Engineering, Weifang University, Weifang 261061, China

**Keywords:** visual sensors, image retrieval, hybrid histogram descriptor, perceptually uniform histogram, motif co-occurrence histogram

## Abstract

Currently, visual sensors are becoming increasingly affordable and fashionable, acceleratingly the increasing number of image data. Image retrieval has attracted increasing interest due to space exploration, industrial, and biomedical applications. Nevertheless, designing effective feature representation is acknowledged as a hard yet fundamental issue. This paper presents a fusion feature representation called a hybrid histogram descriptor (HHD) for image retrieval. The proposed descriptor comprises two histograms jointly: a perceptually uniform histogram which is extracted by exploiting the color and edge orientation information in perceptually uniform regions; and a motif co-occurrence histogram which is acquired by calculating the probability of a pair of motif patterns. To evaluate the performance, we benchmarked the proposed descriptor on RSSCN7, AID, Outex-00013, Outex-00014 and ETHZ-53 datasets. Experimental results suggest that the proposed descriptor is more effective and robust than ten recent fusion-based descriptors under the content-based image retrieval framework. The computational complexity was also analyzed to give an in-depth evaluation. Furthermore, compared with the state-of-the-art convolutional neural network (CNN)-based descriptors, the proposed descriptor also achieves comparable performance, but does not require any training process.

## 1. Introduction

In the past decades, affordable visual sensor equipment (e.g., surveillance cameras, smart phones, digital cameras and camcorders) has become widespread in our daily lives. Due to the growing number of images collected from these visual sensors, how to accurately and quickly retrieve the image-of-interest has become a hot topic [1,2,3,4,5,6]. Compared with text-based image retrieval (TBIR), content-based image retrieval (CBIR) is widely considered as an effective and efficient technology that not only extracts low-level visual cues (e.g., color, shape and texture) automatically, but also bridges high-level semantic comprehension. Until now, the feature representation descriptors, such as independent feature descriptor and fusion-based feature descriptor, have been increasing and developing in the CBIR community.

Color information plays an important role in the feature representation. Currently, color moment [7], color set [8], color coherence vector [9], color correlogram [10] and color histogram [11,12,13,14,15,16,17,18] have been developed for color feature representation continuously. In [11], the color layout descriptor (CLD), scalable color descriptor (SCD), color structure descriptor (CSD) and dominant color descriptor (DCD) are constructed as the color feature descriptors. Subsequently, in [12,13,14,15], a series of equal-interval color quantization models are used for the extraction of color histograms. Recently, in [16], Bayesian Information Criterion (BIC), Expectation Maximization (EM) and Gaussian Mixture Models (GMM) are integrated into a universal color quantization framework. More recently, in [17,18], the combined color histogram is proposed for color feature representation. However, the above methods are confined to quantizing the range of different color channels, and a few consider the color probability distribution of different color channels. In addition, several methods (e.g., Fourier transforms [19], moment invariant [20] and edge orientation detection [13,14,15,21,22,23,24,25]) have been developed for shape-based representation. In [21], edge orientation detection is equipped with different gradient operators for the orientation information computation on grey-scale images. With the appearance of color images, in [13,14,15], a series of edge detection and quantization strategies is applied to capture the geometry and orientation information from color images in different color spaces. In [22,23,24,25], a class of local edge orientation detection descriptors is developed for edge orientation histogram extraction. In short, edge orientation detection and quantization are widely considered as the effective and correct approaches that not only achieve stable performances but also exploit the geometry and orientation information with less computational complexity.

Along other research lines, many strategies [17,18,26,27,28,29] have been designed to represent textural features. For example, the local binary pattern (LBP) [26] is first proposed to code the center pixel and its neighborhood pixels as a binary label in eight directions. Later, the LBP is extended to the local extrema pattern (LEP) [17], which computes the index values between the center pixel and its eight neighbors in four directions. Afterwards, the LEP is modified to the local extrema co-occurrence pattern (LEcP) [18], which reveals the relationship of mutual occurrence patterns in the V channel of the HSV color space. Furthermore, the concept of texton or motif [27] is first defined to analysis the elements of texture perception and their interactions. Recently, a grey-level co-occurrence matrix (GLCM) [28] is treated as a co-occurrence-based relation descriptor that computed the occurrence frequencies of a pair of grey-pixels. More recently, the motif co-occurrence matrix (MCM) [29] is defined as a 3D matrix, in which six motif patterns are designed to calculate the probability of a pair of motif patterns in a pre-defined direction. However, using six motif patterns is incomplete, because the perceptually uniform motif patterns are not further discussed and analyzed.

Although the above-mentioned methods have proven to be effective, independent feature descriptors are inadequate to meet the demands of feature representation. Many studies have proven that fusion-based descriptors are more powerful than independent feature descriptors. In [13,14,15], the color histogram and the edge orientation histogram are treated as a pair of mutual information descriptors, calculated by a color difference operator. In [17], the color histogram is combined with the local extrema pattern histogram used for object tracking in the RGB color space. In [18], the local extrema co-occurrence pattern (LEcP) is transformed into an independent feature vector; then, LEcP is combined with the joint color histogram for feature representation. Again, in [30], a multi-channel decoded local binary pattern (mdLBP) and a multi-channel adder local binary pattern (maLBP) are simultaneously constructed by combining three LBP maps, which are calculated in the RGB color space. Recently, in [31], the local neighborhood difference pattern (LNDP) and the LBP is explored to capture local intensity difference information for the natural and texture image retrieval. In [32], Bianconi et al. provided a general framework and taxonomy of color texture descriptors. In [33], Cusano et al. suggested an evaluation of color texture descriptors under large variations of controlled lighting conditions, whereas Qazi et al. investigated pertinent color spaces for color texture characterization [34]. At the same time, in [35], user relevance feedback, feature re-weight and weight optimization are used to further improve the accuracy of image retrieval.

In this study, the main contributions are summarized as follows:

1. We designed the pyramid color quantization model, which is based on the powerful color probability distribution prior in the L*a*b* color space.
2. We constructed the perceptually uniform histogram, which integrates color and edge orientation as a whole by exploiting a color difference operator.
3. We developed the motif co-occurrence histogram in which the perceptually uniform motif patterns are further discussed and analyzed.
4. We proposed the hybrid histogram descriptor that is comprised of the perceptually uniform histogram and the motif co-occurrence histogram.

The remainder of this paper is organized as follows. Preliminaries are introduced in Section 2, and the feature representation is described in Section 3. Experiments and evaluations are presented in Section 4. Section 5 provides conclusions.

## 2. Preliminaries

### 2.1. The Color Space Selection

The selection of the color space is a crucial step before feature representation. In the past decades, several types of color spaces (e.g., RGB, L*a*b*, HSV, CMYK, YUV and HSI) have been widely used for CBIR. Among them, the RGB is recognized as one of the most popular color spaces. It is derived from three colors of light, namely, red (R), green (G) and blue (B) [36]. Nevertheless, its disadvantages are often ignored: (1) the redundancy between blue and green; (2) the missing yellow between red and green; and (3) the non-uniform perception of human eye. Consequently, Hering defined the L*a*b* color space, which includes three pairs of color channels consisting of the white–black pair of the L* channel (ranging from 0 to 100), the yellow–blue pair of the a* channel (ranging from −128 to +127), and the red–green pair of the b* channel (ranging from −128 to +127) [37]. Compared with the RGB, the advantages of the L*a*b* color space are summarized as follows: (1) the L*a*b* remedies the redundant and missing information of the RGB; (2) it conforms to human eye’s perception mechanism; and (3) it provides excellent decoupling between intensity (represented by the L* channel) and color (represented by the a* and b* channels) [38]. Therefore, our scheme transforms all images from RGB to L*a*b* color space before the feature representation stage. The details of this transformation are defined using standard RGB to L*a*b* transformations as follows [15,39]:

(1) $$\left\{ \begin{array}{ll} L*=116{\left(\frac{Y}{Y_{n}}\right)}^{1/3}-16 & \mathrm{for}\frac{Y}{Y_{n}}>0.08856\\ L*=903.3{\left(\frac{Y}{Y_{n}}\right)}^{1/3} & \mathrm{for}\frac{Y}{Y_{n}}\leq 0.08856 \end{array}\right.,$$

(2) $$a*=500(f(\frac{X}{X_{n}})-f(\frac{Y}{Y_{n}})),$$

(3) $$b*=500(f(\frac{X}{X_{n}})-f(\frac{Y}{Z_{n}})),$$

with

(4) $$\left\{ \begin{array}{ll} f(u)=u^{1/3} & \mathrm{for}u>0.08856\\ f(u)=7.78u+\frac{Y}{Y_{n}} & \mathrm{for}u\leq 0.08856 \end{array}\right.,$$

where

(5) $$\left[\begin{array}{l} X\\ Y\\ Z \end{array}\right]=\left[\begin{array}{l} \begin{array}{ccc} 0.412453 & 0.357580 & 0.180423 \end{array}\\ \begin{array}{ccc} 0.212671 & 0.715160 & 0.072169 \end{array}\\ \begin{array}{ccc} 0.019334 & 0.119193 & 0.950227 \end{array} \end{array}\right]\left[\begin{array}{l} R\\ G\\ B \end{array}\right],$$

where *X_n_*, *Y_n_* and *Z_n_* are the values of *X*, *Y* and *Z* for the illuminant and [*X_n_, Y_n_*, *Z_n_*] = [0.950450, 1.000000, 1.088754] in accordance with illuminant D65 [15].

### 2.2. Probability Distribution Prior in L*a*b* Color Space

**Figure 1:**
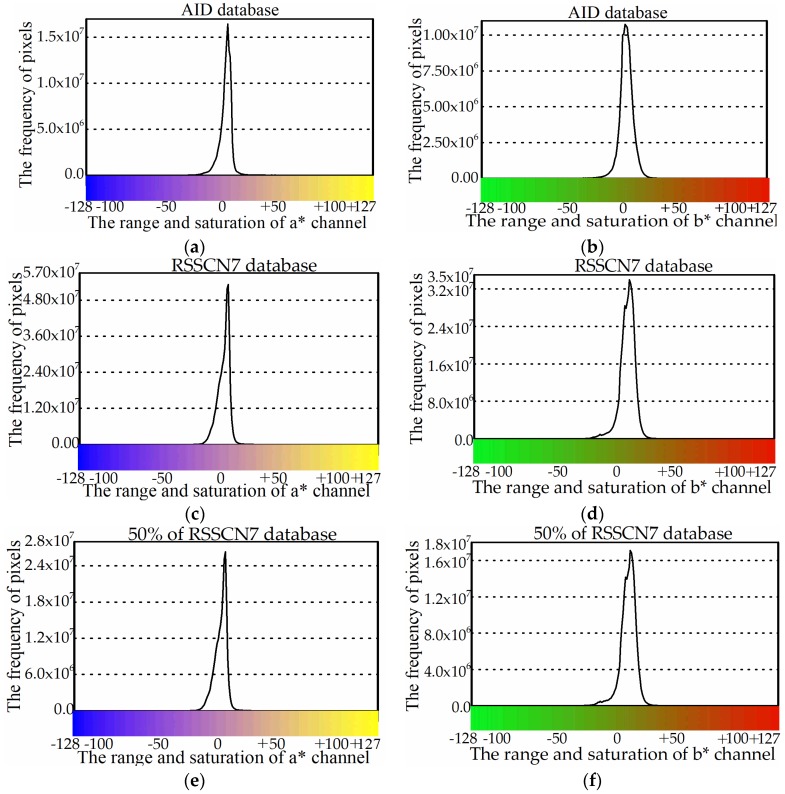
The frequency of pixels over different databases: (**a**,**b**) AID; (**c**,**d**) RSSCN7; and (**e**,**f**) 50% of RSSCN7.

In the previous color quantization models [12,13,14,15,17,18], three color channels are uniformly mapped into the fixed intervals. However, during the process of quantization, these models lose some useful color information. Hence, reducing the loss of the useful color information is a serious concern. Inspired by this motivation, we have explored and summarized the color probability distribution of the a* and b* channels in different image databases. The example of the AID image database [40] is shown in Figure 1a,b. The frequency of pixels mainly focuses on the center region of the a* and b* channels.

To verify the validity of this prior knowledge, we calculated the color probability distribution statistics of the a* and b* channels on hundreds of image databases. The results show that the proposed prior is stable and consistent. Even if an image database has been changed, the property of the color probability distribution prior is still fairly consistent. For example, the color probability distribution of the a* and b* channels in the RSSCN7 [41] dataset and its subset (50% of the RSSCN7 dataset) is shown in Figure 1c–f. Obviously, there is almost no change between RSSCN7 and its subset, except for the pixel frequency.

## 3. Feature Representation

### 3.1. Perceptually Uniform Histogram

#### 3.1.1. Pyramid Color Quantization Model

**Figure 2:**
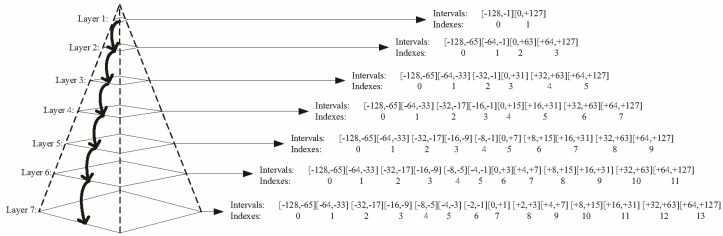
Pyramid color quantization model.

Inspired by the above prior knowledge, we designed a novel pyramid color quantization model (as shown in Figure 2), in which every layer represents a quantized scheme (including a group of intervals and indexes). The original range (−128, +127) of a* or b* is first projected into two equal intervals in Layer 1, and the indexes of two intervals are flagged as 0 and 1 from left to right, correspondingly. Then, considering the pixels focus on the middle, two middle intervals from Layers 2–7 are split into four equal intervals from the up-layer to down-layer until two middle intervals cannot be split in Layer 7. Finally, the remaining intervals are copied from the up-layer to down-layer, sequentially. In this manner, we refine and retain the color information in the middle of the a* or b* channels effectively. We define the quantization layer of the a* and b* channels as *Y*_a*_ and *Y*_b*_, where *Y*_a*_, *Y*_b*_ ∈ {1, 2, …, 7}, and the indexes are denoted as *Ỹ*_a*_ and *Ỹ*_b*_, *Ỹ*_a*_ ∈ {0, 1, …, *Ÿ*_a*_} and *Ỹ*_b*_ ∈ {0, 1, …, *Ÿ*_b*_}, where *Ÿ*_a*_ = 2*Y*_a*_ − 1 and *Ÿ*_b*_ = 2*Y*_b*_ − 1, respectively.

In addition, considering the human visual intensity perception mechanism in [5], the L* channel is quantized into three intervals (0, +25), (+26, +75) and (+76, +100). We define the quantization layer of the L* channel as *Y*_L*_, where *Y*_L*_ = 1, and the index is flagged as *Ỹ*_L*_, *Ỹ*_L*_ ∈ {0, 1, …, *Ÿ*_L*_}, where *Ÿ*_L*_ = 2*Y*_L*_. In summary, combining the indexes of the L*, a* and b* channels, the color map of an image *f*(*x*, *y*) is defined as *C*(*x*, *y*), and the index is flagged as $\tilde{C}$, $\tilde{C}\in$ {0, 1, …, $\hat{C}$}, where $\hat{C}$ = 2*Y*_a*_ × 2*Y*_b*_ × 3 − 1.

#### 3.1.2. Perceptually Uniform Histogram Definition

The Gestalt Psychology Theory elucidates that the human visual perception mechanism tends to group elements into a local region where the elements share a homologous or approximate property [42]. Based on this theoretical foundation, perceptually uniform regions can be described as a certain visual feature space in which visual elements have the same rule (e.g., color and edge orientation). For the visual feature space *Ĩ*, an element *ξ* and its neighborhoods *ξ_g_* within *Ĩ* are flagged as *Ĩ*(*ξ*) and *Ĩ*(*ξ_g_*). Mathematically, the discrimination function *φ*(·) is formulated as follows:

(6) $$\varphi (\tilde{I}(\xi ),\tilde{I}(\xi_{g}))=\left\{ \begin{array}{l} \begin{array}{cc} 1, & \begin{array}{c} \tilde{I}(\xi )=\tilde{I}(\xi_{g}) \end{array} \end{array}\\ \begin{array}{cc} 0, & \tilde{I}(\xi )\neq \tilde{I}(\xi_{g}) \end{array} \end{array}\right.,g\in \{1,2,\ldots ,\ddot{N}\},$$

where $\ddot{N}$ represents the number of neighborhoods. If *φ*(*Ĩ*(*ξ*), *Ĩ*(*ξ_g_*)) = 1, $\tilde{I}$ *Ĩ*(*ξ_g_*) belongs to the perceptually uniform region; if *φ*(*Ĩ*(*ξ*), *Ĩ*(*ξ_g_*)) = 0, $\tilde{I}$(*ξ_g_* does not belong to the perceptually uniform region.

With subject to the perceptually uniform region, we construct the perceptually uniform histogram by exploiting the color difference operator [15,43,44] between the color and edge orientation. Herein, given an image *f*(*x*, *y*), the edge orientation map *O*(*x*, *y*) is first extracted by using the Prewitt operator, due to its advantages of extracting the geometry and boundary information from the observed content. Then, experimentally, the edge orientation value is quantized uniformly into four bins to construct the edge orientation map *O*(*x*, *y*) because it is time consuming and unnecessary to consider all edge orientation values. Finally, the edge orientation map *O*(*x*, *y*) and the color map *C*(*x*, *y*) are divided into the overlapping 3 × 3 windows in which the central pixel is flagged as (*x*, *y*) and its eight neighbors are flagged as (*x_g_*, *y_g_*), g∈{1,2,…,8}. The perceptually uniform histogram (PUH) is defined as follows:

(7) $$PUH^{colour}(O(x,y))\;\;\;\;=\;\;\;\sum_{g=1}^{8}\sqrt{\sum_{\psi \in L*,a*,b*}{\left(\Delta f_{\psi }\right)}^{2}}\;sub.t.\;\varphi (C(x,y),C(x_{g},y_{g}))=1,$$

(8) $$PUH^{ori}(C(x,y))\;\;\;\;=\;\;\;\sum_{g=1}^{8}\sqrt{\sum_{\psi \in L*,a*,b*}{\left(\Delta f_{\psi }\right)}^{2}}\;sub.t.\;\varphi (O(x,y),O(x_{g},y_{g}))\;=\;1,$$

where ∆*f* represents the color differences among the central pixel (*x*, *y*) and its eight neighbors (*x_g_*, *y_g_*) in ψ channels, ψ∈L*,a*,b*. The feature vector length of *PUH^color^*(*O*(*x*, *y*)) and *PUH^ori^*(*C*(*x*, *y*)) are 4 and 2*Y*_a*_ × 2*Y*_b*_ × 3, respectively. For an image dataset *D*, the fitness quantization layers of *Y*_a*_ and *Y*_b*_ are computed depending upon the retrieval accuracy score Acc(*D*|*Y*_a*_, *Y*_b*_). This procedure is expressed as the maximization problem as follows:

(9) $$\underset{Y_{a*},Y_{b*}}{\max}\mathrm{Acc}(D|Y_{a*},Y_{b*}),Y_{a*},Y_{b*}\in \{1,2,\ldots ,7\},$$

We present the detailed evaluation of different color quantization layers of *Y*_a*_ and *Y*_b*_ in Section 4.4.

### 3.2. Motif Co-Occurrence Histogram

The perceptually uniform histogram only extracts the color and edge orientation information, but the texture information is ignored to some extent. Fortunately, the motif pattern, which depicts the texture information by the pre-defined spatial structure model, can remedy this shortcoming.

#### 3.2.1. Motif Patterns

**Figure 3:**
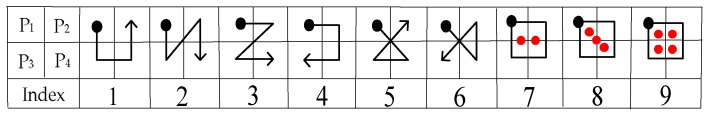
Nine types of motif patterns.

The motif co-occurrence matrix (MCM) is investigated in [29] where the first six types of motif patterns shown in Figure 3, starting from the top-left point P1, are generated because they represent a completed set of space filling curves. However, using merely six motif patterns is insufficient because the perceptually uniform motif patterns (PUMP) are ignored.

To depict the consistency of spatial structure information, we propose three perceptually uniform motif patterns into which all types of perceptually uniform motif patterns are separated based on the number of equal pixels. Combining the previous six motif patterns, nine motif patterns are obtained, as shown in Figure 3, in which the red dots represent the number of equal pixels in the motif patterns 7, 8 and 9.

#### 3.2.2. Motif Co-Occurrence Histogram Definition

**Figure 4:**
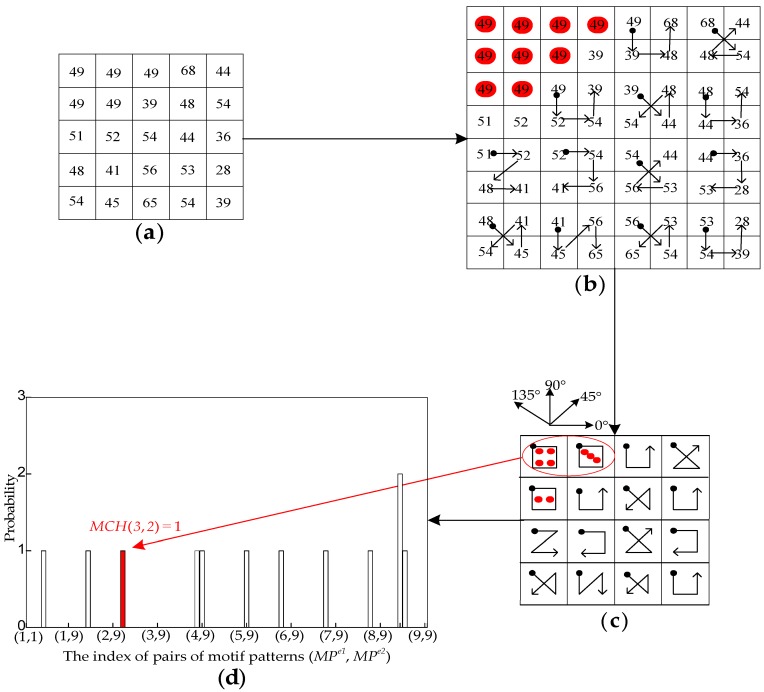
Schematic diagram of the motif co-occurrence histogram: (**a**) a 5 × 5 mini-numerical map; (**b**) the overlapping 2 × 2 grids of (**a**); (**c**) the motif map; and (**d**) the motif co-occurrence histogram.

Since the L*a*b* color space provides excellent decoupling between intensity (represented by the L* channel) and color (represented by the a* and b* channels) [38], the L* channel is applied to extract the motif co-occurrence histogram. For simplicity, a 5 × 5 mini-numerical map in Figure 4a is adopted to illustrate the proposed method. In our scheme, each pixel (apart from the lower and right boundary pixels) in the map is divided into the overlapping 2 × 2 grids in Figure 4b. Then, each grid is transformed into a motif pattern with the minimized local gradient to obtain the motif map shown in Figure 4c, which is used to calculate the motif co-occurrence histogram shown in Figure 4d. For example, the red circle in Figure 4c is a pair of motif patterns, indexed as (3, 2), in the 0° direction, corresponding to the red bar “*MCH*(3, 2) = 1” in the motif co-occurrence histogram in Figure 4d. Mathematically, the probability of co-occurrence of a pair of motif patterns is expressed as follows:

(10) $$MCH(MP^{e1},MP^{e2})=\mathrm{Pr}\{M(i,j)=MP^{e1},M(i,j+1)=MP^{e2}\},$$

where Pr is the probability of co-occurrence of a pair of motif patterns corresponding to (i,j) and its neighbor (i,j+1) within the motif map *M*(*x*, *y*). *MP^e^*^1^ and *MP^e^*^2^ represent the indexes of a pair of motif patterns, where *MP^e1^*, *MP^e2^* ∈{1,2,…,9}. The feature vector length of the motif co-occurrence histogram is 81. We will perform the detailed evaluation of different motif co-occurrence schemes between the motif co-occurrence matrix [29] and the proposed motif co-occurrence histogram in Section 4.5.

### 3.3. Hybrid Histogram Descriptor Definition

It is widely recognized that an image possesses a rich semantic content that goes beyond the description by its metadata [2]. Hence, it is necessary to take a fusion-based feature descriptor into account because it can integrate the merits of the subjective aspects of image semantics. From this point of view, the hybrid histogram descriptor (HHD) is proposed by concatenating the perceptually uniform histogram and the motif co-occurrence histogram, and it is expressed as follows:

(11) HHD=[PUH,MCH],

We present the detailed evaluation of the proposed descriptors among the perceptually uniform histogram, the motif co-occurrence histogram and the hybrid histogram descriptor in Section 4.6.

## 4. Experiments and Discussion

### 4.1. Distance Metric

The distance metric serves as an important step to measure the feature vector dissimilarity. In the CBIR framework, the query image and database images are converted into feature vectors in the form of histogram descriptors, and they are sent to the distance measure for measuring the dissimilarity. In this paper, the Extended Canberra Distance [15,32] is used, and it is defined as follows:

(12) $$T(D,Q)=\sum_{\mu =1}^{K}\frac{\left|D_{\mu }-Q_{\mu }\right|}{\left|D_{\mu }+l_{D}\right|+\left|Q_{\mu }+l_{Q}\right|},$$

where *Q*, *D*, *K*, and *T* represent the query image, the database image, the feature vector dimension, and the distance metric result, respectively, where $l_{D}=\sum_{\mu =1}^{K}D_{\mu }/K$ and $l_{Q}=\sum_{\mu =1}^{K}Q_{\mu }/K$.

### 4.2. Evaluation Criteria

The final goal of image retrieval is to search a set of target images from the image database [35]. For a query image *I_Q_* and a database image *I_D_*, the precision (*Pre*) and recall (*Rec*) values are given as follows:

(13) $$Pre\;\;\;\;=\;\;\;\frac{1}{N_{\sigma }}\sum_{D=1}^{N_{\sigma }}\varsigma (\vartheta (I_{Q}),\vartheta (I_{D}))\times 100\%,$$

(14) $$Rec\;\;\;\;=\;\;\;\frac{1}{N_{\tau }}\sum_{D=1}^{N_{\tau }}\varsigma (\vartheta (I_{Q}),\vartheta (I_{D}))\times 100\%,$$

(15) $$\varsigma (\vartheta (I_{Q}),\vartheta (I_{D}))=\left\{ \begin{array}{l} \begin{array}{cc} 1, & \mathrm{if}\begin{array}{c} \vartheta (I_{Q})=\vartheta (I_{D}) \end{array} \end{array}\\ \begin{array}{cc} 0, & \mathrm{otherwise} \end{array} \end{array}\right.,$$

where ϑ(⋅), $N_{\sigma }$, and $N_{\tau }$ represent the image category information, the number of retrieved images, and the number of images in each category, respectively. The discrimination function ς(⋅) is used to determine the category information between the query image and the database images. In the experiments, to guarantee accuracy and reproducibility, all images were chosen as the query image. Referring to the parameter setting in [30,32], the number of retrieved images was set to 10. For ETHZ-53 [45], the number of retrieved images was set to 5.

Further, for *N* query images, the average precision rate (APR) and average recall rate (ARR) values are defined as follows:

(16) $$APR\;\;=\;\;\;\frac{\sum_{n=1}^{N}Pre(n)}{N}\times 100\%,$$

(17) $$ARR\;\;=\;\;\;\frac{\sum_{n=1}^{N}Rec(n)}{N}\times 100\%,$$

where n is the *n*th query image.

Furthermore, considering the order of the retrieved images, the precision–recall curve denotes an auxiliary evaluation criterion that measures the dynamic precision with the threshold recall. Mathematically, the precision–recall curve is formulated as follows:

(18) $$PR\;\;(\chi )=\;\;\;\frac{N_{\tau }}{N_{\chi }}\cdot \chi \times 100\%,$$

where *N_τ_* and *N_χ_* represent the number of images in each category, and the total number of the shown images at the recall of *χ*, *χ* ∈ {1, 2, …, *N*_σ_ − 1}. A higher precision–recall curve indicates a more accurate retrieval performance.

### 4.3. Image Databases

Extensive experiments were conducted on five benchmark databases, including two remote sensing image databases (RSSCN7 and AID), two textural image datasets (Outex-00013 and Outex-00014), and one object image database (ETHZ-53). The details of these datasets are summarized as follows:

1. RSSCN7 database

**Figure 5:**
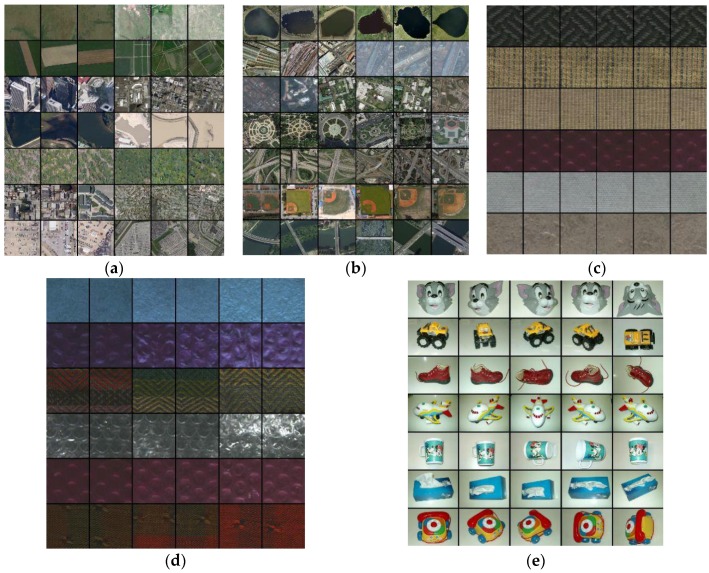
Some sample images from different databases: (**a**) RSSCN7; (**b**) AID; (**c**) Outex-00013; (**d**) Outex-00014; and (**e**) ETHZ-53.

The RSSCN7 [41] is a publicly available remote sensing dataset produced by different remote imaging sensors. It consists of seven land-use categories, such as industrial region, farm land, residential region, parking lot, river lake, forest and grass land. For each category, there are 400 images with size of 400 × 400 in JPG format. Some sample images are shown in Figure 5a, in which each row represents one category. Note that there are images with rotation and resolution differences in the same category. Thus, the RSSCN7 dataset can not only verify the effective of the proposed descriptor but also inspect the robustness of different rotations and resolutions. The RSSCN7 dataset can be downloaded from https://www.dropbox.com/s/j80iv1a0mvhonsa/RSSCN7.zip?dl=0.

2. AID database

The aerial image dataset (AID) [40] is also a publicly available large-scale remote sensing dataset produced by different remote imaging sensors. It contains 10,000 images in 30 categories, for example, airport, bare land, meadow, beach, park, bridge, forest, railway station, and baseball field. Each category includes different numbers of images varying from 220 to 420 with size of 600 × 600 in JPG format. Some sample images are shown in Figure 5b, in which each row is one category. Similar to RSSCN7, there are images with rotation and resolution differences in the same category. The AID dataset can be downloaded from http://www.lmars.whu.edu.cn/xia/AID-project.html.

3. Outex-00013

The Outex-00013 [46] is a publicly available color texture dataset produced by an Olympus Camedia C-2500 L digital camera. It contains 1360 images in 68 categories, for example, wool, fabric, cardboard, sandpaper, natural stone and paper. Each category includes 20 images, each with size of 128 × 128 in BMP format. Some sample images from Outex-00013 are shown in Figure 5c, in which each row represents one category. There is no difference in the same category. The Outex-00013 dataset can be downloaded from http://www.outex.oulu.fi/index.php?page=classification.

4. Outex-00014

The Outex-00014 [46] is also a publicly available color texture dataset produced by an Olympus Camedia C-2500 L digital camera. It contains 4080 images in 68 categories, for example, wool, fabric, cardboard, sandpaper, natural stone, and paper. Each category includes 20, each with size of 128 × 128 images in BMP format. Some sample images from Outex-00014 are shown in Figure 5d, in which each row represents one category. All images are produced under three different illuminants: the 4000 K fluorescent TL84 lamp, the 2856 K incandescent CIE A and the 2300 K horizon sunlight. The Outex-00014 dataset can also be downloaded from http://www.outex.oulu.fi/index.php?page=classification.

5. ETHZ-53

The ETHZ-53 [45] is a publicly available object dataset collected by a color camera. It contains 265 images in 53 objects, such as cup, shampoo, vegetable, fruit, and car model. Each object includes 5 images, each with size of 320 × 240 in BNG format. Some sample images are shown in Figure 5e, in which each row represents one category. Note that each object is with 5 different angles. The ETHZ-53 dataset can be downloaded from http://www.vision.ee.ethz.ch/en/datasets/.

### 4.4. Evaluation of Different Color Quantization Layers

Table 1, Table 2, Table 3, Table 4 and Table 5 show the average precision rate (APR) of the proposed descriptor on the RSSCN7, AID, Outex-00013, Outex-00014 and ETHZ-53 datasets under different color quantization layers of *Y*_a*_ and *Y*_b*_, where *Y*_a*_, *Y*_b*_ ∈ {1, 2, …, 7}. Bold values highlight the best values. As reported in Table 1 and Table 2, i when *Y*_a*_ = 6 and *Y*_b*_ = 5, the HHD achieves the best APR = 79.57% on RSSCN7 and the best APR = 58.13% on AID, respectively. As documented in Table 3 and Table 4, when *Y*_a*_ = 6 and *Y*_b*_ = 2, the HHD achieves the best APR = 84.21% on Outex-00013 and the best APR = 82.82% on Outex-00014, respectively. As listed in Table 5, when *Y*_a*_ = 5 and *Y*_b*_ = 6, the HHD achieves the best APR = 97.89% on ETHZ-53. In addition, we can also see that the simplest color quantization scheme (e.g., *Y*_a*_ = 1 and *Y*_b*_ = 1) does not lead to the lowest APR on RSSCN7 and Outex-00013, and the most refined color quantization scheme (e.g., *Y*_a*_ = 7 and *Y*_b*_ = 7) does not guarantee the highest APR. This phenomenon demonstrates that it is necessary to adaptively select the fitness quantization layers of *Y*_a*_ and *Y*_b*_. Depending upon the retrieval accuracy score, the fitness quantization layers of *Y*_a*_ and *Y*_b*_ will be used in the following experiments*.*

### 4.5. Evaluation of Different Motif Co-Occurrence Schemes

Table 6 shows the average precision rate (APR) and average recall rate (ARR) values on the RSSCN7, AID, Outex-00013, Outex-00014 and ETHZ-53 datasets by using the motif co-occurrence matrix (MCM) and the motif co-occurrence histogram (MCH), respectively. Bold values highlight the best values. In Table 6, the {APR, ARR} of MCH greatly outperforms MCM by {18.14%, 0.45%} on RSSCN7, {15.21%, 0.47%} on AID, {41.75%, 20.87%} on Outex-00013 and {24.63%, 12.32%} on Outex-00014. One possible reason is that MCH takes three perceptually uniform motif patterns. Based on the above results, it can be concluded that MCH is more effective than MCM.

### 4.6. Evaluation of the Proposed Descriptors

Table 7 shows the average precision rate (APR) and average recall rate (ARR) values on the RSSCN7, AID, Outex-00013, Outex-00014 and ETHZ-53 datasets by using the motif co-occurrence histogram (MCH), the perceptually uniform histogram (PUH) and the hybrid histogram descriptor (HHD). Bold values highlight the best values. As listed in Table 7, the {APR, ARR} of HHD outperforms MCH by {15.47%, 0.39%} on RSSCN7, by {20.09%, 0.61%} on AID, by {15.61%, 7.80%} on Outex-00013, by {41.91%, 20.95%} on Outex-00014 and by {49.51%, 49.51%} on ETHZ-53. Meanwhile, it can also be observed that the {APR, ARR} of HHD outperforms PUH by {7.35%, 0.18%} on RSSCN7, by {7.09%, 0.21%} on AID, by {4.81%, 2.40%} on Outex-00013, by {6.68%, 3.34%} on Outex-00014, and by {0.08%, 0.08%} on ETHZ-53, respectively. The main reason is that HHD integrates the merits of PUH and MCH effectively. Based on the above results, it can be asserted that HHD performs better than MCH and PUH significantly.

### 4.7. Comparison with Other Fusion-Based Descriptors

To illustrate the effectiveness and robustness of hybrid histogram descriptor (HHD), it is compared with nine fusion-based feature descriptors and the fusion of the perceptually uniform histogram and motif co-occurrence matrix (flagged as “PUH + MCM”) on the RSSCN7, AID, Outex-00013, Outex-00014 and ETHZ-53 datasets. All comparative methods are detailed as follows:

(1) mdLBP [30]: The 2048-dimensional multichannel adder local binary patterns by combining three LBP maps extracted from the R, G and B channels.
(2) maLBP [30]: The 1024-dimensional multichannel decoded local binary patterns by combining three LBP maps extracted from the R, G and B channels.
(3) CDH [15]: The 90-dimensional color histogram obtained by quantizing the L*a*b* color space and the 18-dimensional edge orientation histogram extracted from the L*a*b* color space.
(4) MSD [14]: The 72-dimensional color histogram obtained by quantizing the HSV color space and the 6-dimensional edge orientation histogram extracted from the HSV color space.
(5) LNDP + LBP [31]: The 512-dimensional local neighborhood difference pattern extracted from the grey-scale space and the 256-dimensional LBP extracted from the grey-scale space.
(6) MPEG-CED [25]: The 256-dimensional color histogram descriptor (CHD) extracted from the RGB color space, and the 5-dimensional edge histogram extracted from the HSV color space.
(7) Joint colorhist [12]: The 512-dimensional color histogram obtained by combining the quantized R, G and B channels.
(8) OCLBP [47]: The fusion of the 1536-dimensional opponent color local binary patterns extracted from the RGB color space.
(9) IOCLBP [46]: The fusion of the 3072-dimensional improved opponent color local binary patterns extracted from the RGB color space.
(10) PUH + MCM: The fusion of the 148/364-dimensional perceptually uniform histogram (PUH) extracted from the L*a*b* color space and the 36-dimensional motif co-occurrence matrix (MCM) extracted from the grey-scale space.
(11) HHD: The fusion of the 148/364-dimensional perceptually uniform histogram (PUH) and the 81-dimensional motif co-occurrence histogram (MCH) extracted from the L* channel.

Quantitative and Qualitative performance valuations are performed from the following seven perspectives: the average precision rate (APR) value, the average recall rate (ARR) value, the average precision rate versus number of top matches (APR vs. NTM), the average recall rate versus number of top matches (ARR vs. NTM), the top-10 retrieved images, the precision–recall curve and the computational complexity. Meanwhile, the robustness of rotation, illumination and resolution is also illustrated in our comparative experiments. To guarantee the accuracy of the experiments, all experiments are performed under the principle of leave-one-out cross-validation.

Table 8 reports the comparisons between the proposed descriptors and the former schemes in terms of average precision rate (APR) and average recall rate (ARR). Bold values highlight the best values. In Table 8, it can be seen that HHD yields the highest APR and ARR compared to all former existing schemes on five datasets. For example, the {APR, ARR} of HHD on RSSCN7 outperforms mdLBP, maLBP, CDH, MSD, LNDP + LBP, MPEG-CED, OCLBP, IOCLBP and PUH + MCM by {6.47%, 0.16%}, {8.69%, 0.22%}, {5.97%, 0.15%} and {11.13%, 0.28%}, {10.11%, 0.25%}, {4.18%, 0.11%}, {6.75%, 0.17%}, {8.87%, 0.24%}, {9.61%, 0.24%} and {5.63%, 0.14%}, respectively. Similarly, more significant values are reported over AID, Outex-13, Outex-14 and ETHZ-53. From these results, the effectiveness of the proposed descriptor is demonstrated by comparing with other fusion-based feature descriptors in terms of APR and ARR. In addition, since there are various rotation and resolution differences on RSSCN7 and AID datasets (see Figure 5a,b), and various illumination differences on Outex-00014 dataset (see Figure 5d), the robustness of the rotation, resolution and illumination is also well illustrated to some extent.

**Figure 6:**
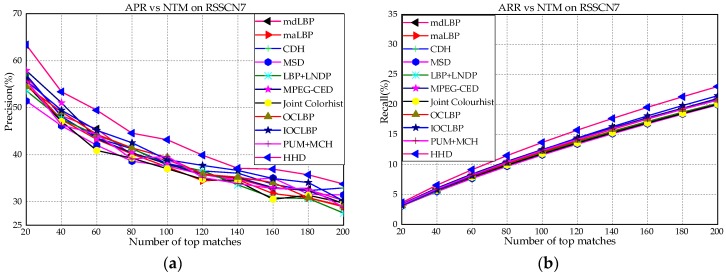

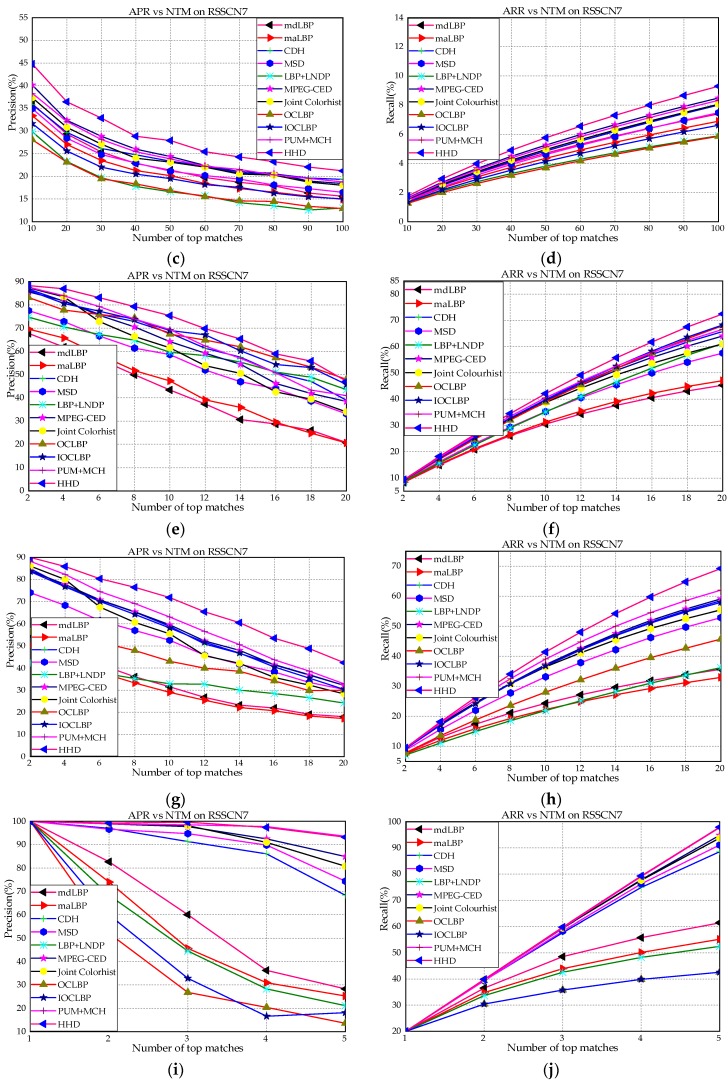
Precision vs. number of top matches (APR vs. NTM) and Recall vs. number of top matches (APR vs. NTM) using different methods over: (**a**,**b**) RSSCN7; (**c**,**d**) AID; (**e**,**f**) Outex-00013; (**g**,**h**) Outex-00014; and (**i**,**j**) ETHZ-53.

Figure 6a–j shows the performance comparison between HHD and existing approaches in terms of average precision rate versus number of top matches (APR vs. NTM) and average recall rate versus number of top matches (ARR vs. NTM). To guarantee the accuracy and reproducibility, the number of top matches is set to 100, 200, 20, 20 and 5 on RSSCN7, AID, Outex-00013, Outex-00014 and ETHZ-53, respectively. In Figure 6a,b, HHD achieves an obviously higher performance than all other fusion-based feature descriptors on RSSCN7. Meanwhile, we also note that the APR vs. NTM and ARR vs. NTM curves of mdLBP, maLBP, CDH, MSD, LNDP + LBP, MPEG-CED, Joint Colorhist, OCLBP, IOCLBP and PUH + MCM are close to one another extremely. The reason is that only seven land-use categories are very challenging to retrieve the targeted images from RSSCN7. As shown in Figure 6c,d, the APR vs. NTM and ARR vs. NTM curves of HHD achieve an obviously higher curvature than all other descriptors on AID. This phenomenon illustrates that the proposed descriptor can acquire better performance on the large-scale dataset. As expected, as shown in Figure 6e–j, HHD still outperforms all other existing descriptors over Outex-00013, Outex-00014 and ETHZ-53, respectively. Specifically, PUM + MCM and HHD are superior to other descriptors on ETHZ-53 obviously. The main reason is that they not only combine the color and edge information, but also integrate the texture information. Based on the above results, the effectiveness of the proposed descriptor is demonstrated by comparing with other fusion-based methods in terms of APR vs. NTM and ARR vs. NTM.

**Figure 7:**
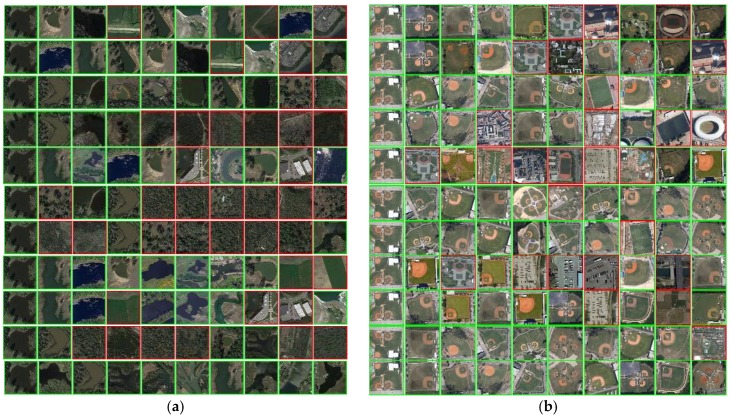

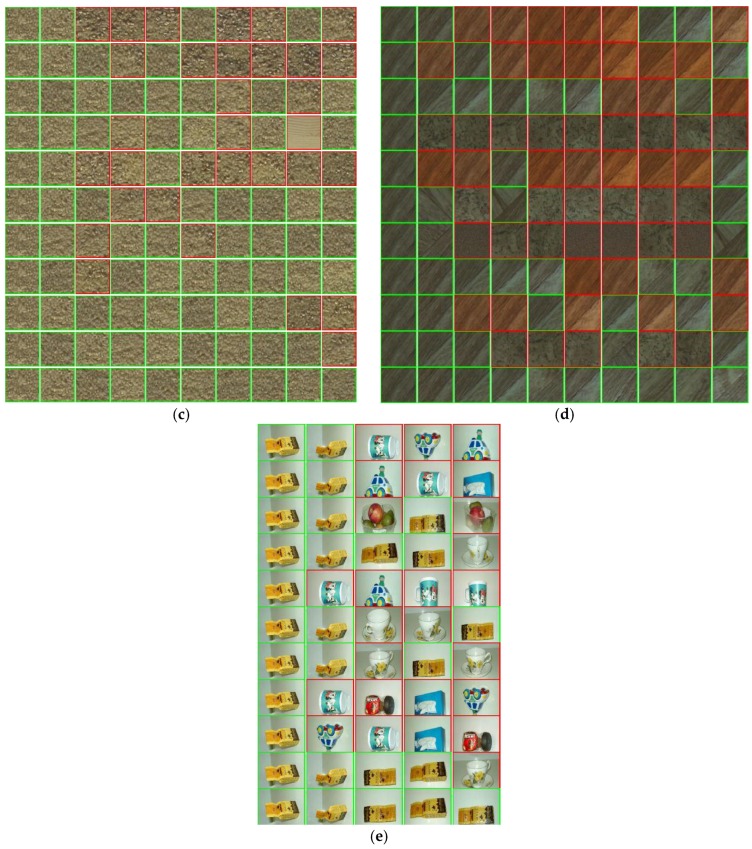
Results of the top-10 retrieved images by considering different query images: (**a**) “River Lake”; (**b**) “Baseball Field”; (**c**) “Rice”; (**d**) “Carpet”; and (**e**) “Paper Bag” using different descriptors (Row 1 using mdLBP, Row 2 using maLBP, Row 3 using CDH, Row 4 using MSD, Row 5 using LNDP + LBP, Row 6 using MPEG-CED, Row 7 using Joint Colorhist, Row 8 using OCLBP , and Row 9 using IOCLBP, Row 10 using PUH + MCM and Row 11 using HHD).

Figure 7a–e shows the performance comparison of the top-10 retrieved images using different methods. The leftmost image in each row of Figure 7a–e is the query image, and the remaining images are a set of retrieved images ordered in ascending order from left to right. For clarity, if a retrieved image owns the same group label as the query, it is flagged as a green frame; otherwise, it is flagged as a red frame. In Figure 7a, there are 7 related images to the query image “River Lake” from RSSCN7 using mdLBP, 8 using maLBP, 8 using CDH, 4 using MSD, 9 using LNDP + LBP, 3 using MPEG-CED, 3 using Joint Colorhist, 8 using OCLBP, 7 using IOCLBP, 4 using PUH + MCM and 10 using HHD. Note that, although the images from “Forest” have a similar color to “River Lake”, leading to the error results by most of the existing schemes, HHD can retrieve the targeted images accurately. In Figure 7b, for the query image “Baseball Field” from AID, the number of targeted images using mdLBP, maLBP, CDH, MSD, LNDP + LBP, MPEG-CED, Joint Colorhist, OCLBP, IOCLBP, PUH + MCM, and HHD descriptors are 7, 7, 9, 6, 5, 9, 5, 8, 9, 9 and 10, respectively. It can be seen that HHD not only displays a better retrieval result than all other descriptors, but also shows the robustness of rotation and resolution differences. In Figure 7c, for the query image “Rice” from Outex-00013, the precision achieved by using mdLBP, maLBP, CDH, MSD, LNDP + LBP, MPEG-CED, Joint Colorhist, PUH + MCM, and HHD descriptors are 40%, 40%, 80%, 70%, 30%, 80%, 80%, 90% and 100%, respectively. In comparison, we can see that although all retrieved images show a similar content appearance, yet HHD still outperforms all other descriptors. In Figure 7d, for the query image “Carpet” from Outex-00014, the precision obtained by using mdLBP, maLBP, CDH, MSD, LNDP + LBP, MPEG-CED, Joint Colorhist, OCLBP, IOCLBP, PUH + MCM, and HHD descriptors are 40%, 30%, 70%, 10%, 30%, 40%, 30%, 70%, 50%, 50% and 100%, respectively. As shown in Figure 7e, for the query image “Paper Bag” from ETHZ-53, HHD still outperforms all other existing descriptors. From the above results, we can conclude that HHD not only depicts the image semantic information with similar textural structure appearance but also discriminates the color and texture differences, effectively. In summary, the effectiveness of the proposed descriptor is demonstrated by comparing with existing approaches in terms of the top-10 retrieved images.

**Figure 8:**
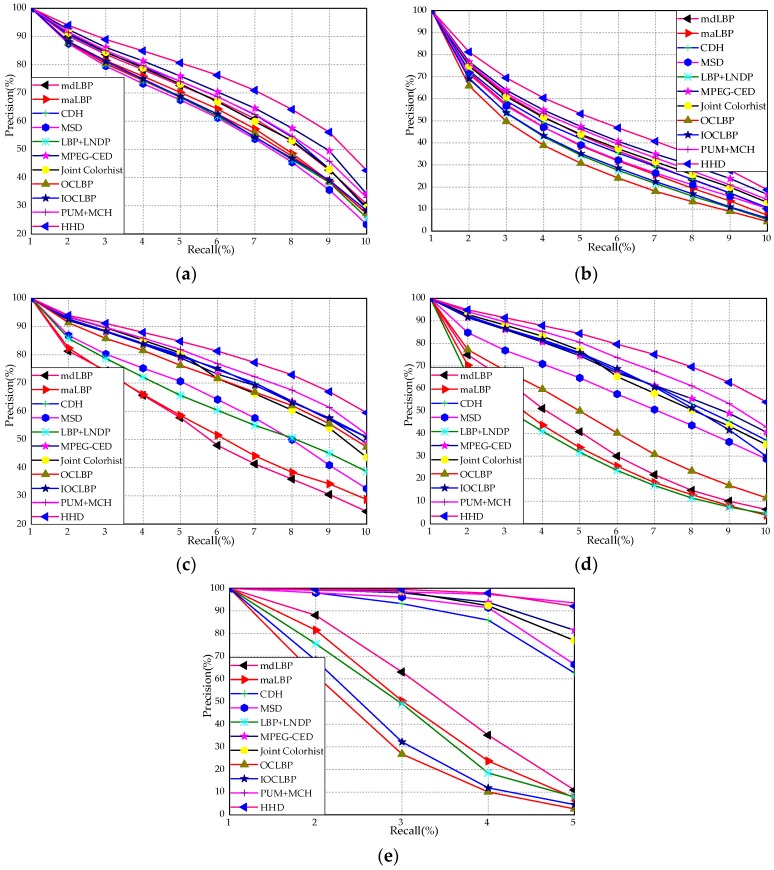
Precision–recall curve of different descriptors over five databases: (**a**) Outex-00013; (**b**) Outex-00014; (**c**) RSSCN7; (**d**) AID; and (**e**) ETHZ-53.

Figure 8a–e shows the performance comparison of the proposed HHD with existing approaches over RSSCN7, AID, Outex-00013 and Outex-00014 in terms of the precision–recall curve. According to Figure 8a,b, it can be observed that the precision–recall curve of HHD is obviously superior to all other fusion-based approaches. According to Figure 8c,d, it can be seen that the precision–recall curve of other fusion-based approaches is inferior to HHD over Outex-00013 and Outex-00014 obviously. Moreover, as shown in Figure 8e, both HHD and PUH + MCM are higher than mdLBP, maLBP, CDH, MSD, LNDP + LBP, OCLBP, IOCLBP, and Joint Colorhist on ETHZ-53. The reasons can be summarized as follows:

(1) Joint Colorhist, mdLBP, maLBP and LNDP + LBP only extract an independent color or texture information.
(2) CDH, MSD and MPEG-CED consider the color and edge orientation information from different channels, while the texture information is ignored.
(3) OCLBP and IOCLBP combine the color and texture information, but the edge orientation information is lost.
(4) Although PUH + MCM integrates the color, edge orientation and texture information as a whole, the perceptually uniform motif patterns are lost.
(5) HHD not only integrates the merits of the color, edge orientation and texture information, but also considers the perceptually uniform motif patterns.

Depending upon the above results and analyses, the effectiveness of the proposed descriptor is demonstrated by comparing with other fusion-based methods in terms of the precision–recall curve.

Table 9 shows the feature vector length, average retrieval time, and memory cost per image of different descriptors to provide an in-depth evaluation of the computational complexity. All experiments are carried out on a computer with Intel Core i7-7700K@4.20 GHz CPU processor, 4 cores active and 16 GB RAM. The feature vector length is compared by dimension (D). The average retrieval time is analyzed by seconds (S). The memory cost per image is measured in kilobytes (KB). Similar to PUM + MCM, the items of 445/229 (D) and 3.48/1.79 (KB) represent HHD with 445 dimensions and 3.48 kilobytes performing retrieval over RSSCN7, AID and ETHZ-53 databases, as well as HHD with 229 dimensions and 1.79 kilobytes performing retrieval over Outex-00013 and Outex-00014 databases. For RSSCN7, AID and ETHZ-53, the feature vector length and the memory cost per image of HHD are inferior to those of MSD, CDH, MPEG-CED and PUM + MCM, while HHD are superior to Joint Colorhist, maLBP, mdLBP, OCLBP, IOCLBP and LNDP + LBP For Outex-00013 and Outex-00014, the feature vector length and the memory cost per image of HHD are worse than MSD, CDH and PUM + MCM, but it is better than MPEG-CED, Joint Colorhist, maLBP, mdLBP, OCLBP, IOCLBP and LNDP + LBP. For the average retrieval time, HHD is more than MSD, CDH, MPEG-CED and PUM + MCM, yet HHD is less than Joint Colorhist, maLBP, mdLBP, OCLBP, IOCLBP and LNDP + LBP. The main reason is that the RSSCN7, AID and ETHZ-53 databases have more complex contents as compared with the Outex-00013 and Outex-00014 image databases. Although HHD does not outperform all other fusion-based descriptors, the usability and practicability of HHD is indicated under the content-based image retrieval framework configuration: adaptive feature vector length, competitive average retrieval time, and acceptable memory cost per image.

### 4.8. Comparison with CNN-Based Descriptors

Apart from the fusion-based descriptors, HHD is also compared with emerging deep neural networks techniques. Referring to the experimental setting in [48], we first extracted the last full-connected layer from the pre-trained CNN model (e.g., VGGM1024 and VGGM128). Then, the extracted feature vectors were L2 normalized. Finally, the normalized feature vectors were sent to perform the distance measure. To guarantee a fair comparison, the number of query images were identically set as all images, and the number of retrieved images were set to 10 on RSSCN7, AID, Outex-00013 and Outex-00014, and 5 on ETHZ-35.

**Figure 9:**
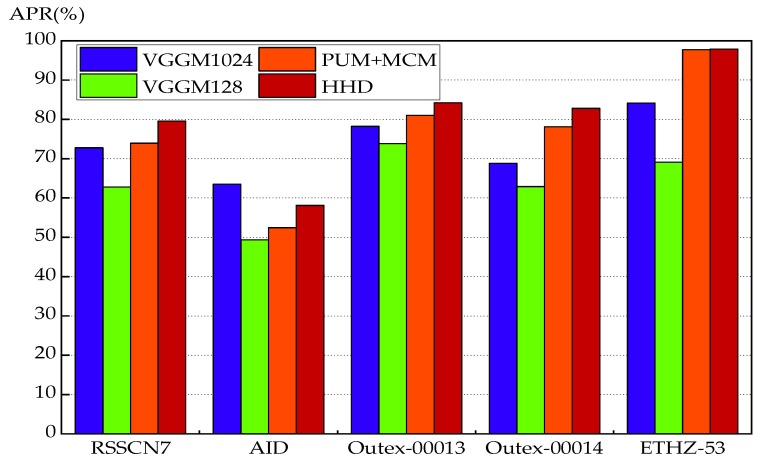
Comparison of the proposed descriptors with the CNN-based schemes over Outex-00013, Outex-00014, RSSCN7, AID and ETHZ-53.

Figure 9 shows the comparisons between the proposed descriptors and the CNN-based schemes. In the case of the RSSCN7, Outex-00013, Outex-00014 and ETHZ-35 datasets, HHD performs better than the VGGM1024 and VGGM128 descriptors, and it achieves the highest performance. Particularly, PUM + MCM also outperforms the VGGM1024 and VGGM128 descriptors on the four datasets. Regarding the AID dataset, HHD is worse than VGGM1024. This makes sense because the pre-trained CNN models which are trained on the large-scale imageset, are suitable for the large-scale AID dataset. In contrast to the CNN-based descriptors, the advantages of HHD can be summarized as follows:

(1) HHD does not require any training process in the feature representation.
(2) The pre-trained CNN-based models have a high memory cost which limits its application.
(3) HHD performs better than the CNN-based descriptors in four datasets out of five.

## 5. Conclusions

In this paper, we propose a fusion method called hybrid histogram descriptor (HHD), which integrates the perceptually uniform histogram and the motif co-occurrence histogram as a whole. The proposed descriptor was evaluated under the content-based image retrieval framework on the RSSCN7, AID, Outex-00013, Outex-00014 and ETHZ-53 datasets. From the experimental results, it can be concluded that the fitness quantization layers of *Y*_a*_ and *Y*_b*_ are computed depending upon the retrieval accuracy score. It is also deduced that the motif co-occurrence histogram (MCH) exhibits significantly higher performance than the motif co-occurrence matrix (MCM). The performance of the proposed descriptor is much improved by confusing the perceptually uniform histogram (PUH) and the motif co-occurrence histogram (MCH). The performance of the proposed descriptor is superior to ten fusion-based feature descriptors in terms of the average precision rate (APR), the average recall rate (ARR), the average precision rate versus number of top matches (APR vs. NTM), the average recall rate versus number of top matches (ARR vs. NTM), and the top-10 retrieved images. Meanwhile, the feature vector length, the average retrieval time, and the memory cost per image were also analyzed to give an in-depth evaluation of the computational complexity. Moreover, compared with the CNN-based descriptors, the proposed descriptor also achieves comparable performance, but does not require any training process.

The increased dimension of the proposed descriptor slows down the retrieval time, which will be addressed in future research, especially using Locality-Sensitive Hashing [49]. Meanwhile, user relevance feedback, feature re-weight and weight optimization will be considered to further improve the accuracy of image retrieval. In addition, we will further investigate the generalization of the proposed method, especially using RawFooT [50] that includes changes in the illumination conditions.

## Figures and Tables

**Table 1:** Average precision rate (APR) of different color quantization layers on RSSCN7.

The Color Quantization Layer for *Y*_a*_	The Color Quantization Layer for *Y*_b*_						
	*Y*_b*_ = 1	*Y*_b*_ = 2	*Y*_b*_ = 3	*Y*_b*_ = 4	*Y*_b*_ = 5	*Y*_b*_ = 6	*Y*_b*_ = 7
***Y*** _a*_ **= 1**	76.65	76.62	76.65	77.18	77.59	77.69	77.56
***Y*** _a*_ **= 2**	76.61	76.59	76.56	77.21	77.59	77.81	77.61
***Y*** _a*_ **= 3**	76.64	76.56	76.45	77.12	77.51	77.81	77.64
***Y*** _a*_ **= 4**	77.32	77.24	77.09	77.42	77.88	78.18	78.11
***Y*** _a*_ **= 5**	78.20	78.21	78.18	78.43	79.05	79.34	79.12
***Y*** _a*_ **= 6**	79.00	79.08	79.10	79.20	**79.57**	79.54	79.26
***Y*** _a*_ **= 7**	78.75	78.90	78.91	78.94	79.26	79.24	78.68

**Table 2:** Average precision rate (APR) of different color quantization layers on AID.

The Color Quantization Layer for *Y*_a*_	The Color Quantization Layer for *Y*_b*_						
	*Y*_b*_ = 1	*Y*_b*_ = 2	*Y*_b*_ = 3	*Y*_b*_ = 4	*Y*_b*_ = 5	*Y*_b*_ = 6	*Y*_b*_ = 7
***Y*** _a*_ **= 1**	53.07	53.19	53.40	55.01	55.75	55.72	55.52
***Y*** _a*_ **= 2**	53.19	53.31	53.54	55.15	55.96	55.90	55.74
***Y*** _a*_ **= 3**	53.30	53.47	53.70	55.19	56.02	55.96	55.81
***Y*** _a*_ **= 4**	54.52	54.65	54.85	56.05	56.74	56.79	56.71
***Y*** _a*_ **= 5**	56.18	56.27	56.35	57.17	57.68	57.79	57.83
***Y*** _a*_ **= 6**	56.83	57.02	57.06	57.81	**58.13**	57.99	57.76
***Y*** _a*_ **= 7**	56.68	56.83	56.91	57.75	57.99	57.71	57.50

**Table 3:** Average precision rate (APR) of different color quantization layers on Outex-00013.

The Color Quantization Layer for *Y*_a*_	The Color Quantization Layer for *Y*_b*_						
	*Y*_b*_ = 1	*Y*_b*_ = 2	*Y*_b*_ = 3	*Y*_b*_ = 4	*Y*_b*_ = 5	*Y*_b*_ = 6	*Y*_b*_ = 7
***Y*** _a*_ **= 1**	83.41	83.52	83.21	82.60	82.28	81.55	81.21
***Y*** _a*_ **= 2**	83.52	83.61	83.23	82.79	82.38	81.73	81.39
***Y*** _a*_ **= 3**	83.54	83.72	83.20	82.99	82.54	81.79	81.31
***Y*** _a*_ **= 4**	83.43	83.55	83.13	82.87	82.44	81.62	81.28
***Y*** _a*_ **= 5**	83.38	83.59	83.10	82.76	82.36	81.54	81.14
***Y*** _a*_ **= 6**	84.11	**84.21**	83.78	83.32	82.84	82.00	81.76
***Y*** _a*_ **= 7**	83.87	83.92	83.65	83.26	82.82	81.84	81.35

**Table 4:** Average precision rate (APR) of different color quantization layers on Outex-00014.

The Color Quantization Layer for *Y*_a*_	The Color Quantization Layer for *Y*_b*_						
	*Y*_b*_ = 1	*Y*_b*_ = 2	*Y*_b*_ = 3	*Y*_b*_ = 4	*Y*_b*_ = 5	*Y*_b*_ = 6	*Y*_b*_ = 7
***Y*** _a*_ **= 1**	79.22	79.33	80.11	81.44	81.60	81.34	81.02
***Y*** _a*_ **= 2**	79.33	79.43	80.20	81.59	81.71	81.49	81.19
***Y*** _a*_ **= 3**	79.36	79.45	80.17	81.62	81.85	81.57	81.21
***Y*** _a*_ **= 4**	80.71	80.76	80.84	81.84	81.92	81.69	81.35
***Y*** _a*_ **= 5**	82.00	82.22	81.99	82.43	82.35	82.06	81.80
***Y*** _a*_ **= 6**	82.71	**82.82**	82.59	82.69	82.56	82.31	82.13
***Y*** _a*_ **= 7**	82.54	82.68	82.59	82.72	82.58	82.35	82.09

**Table 5:** Average precision rate (APR) of different color quantization layers on ETHZ-53.

The Color Quantization Layer for *Y*_a*_	The Color Quantization Layer for *Y*_b*_						
	*Y*_b*_ = 1	*Y*_b*_ = 2	*Y*_b*_ = 3	*Y*_b*_ = 4	*Y*_b*_ = 5	*Y*_b*_ = 6	*Y*_b*_ = 7
***Y*** _a*_ **= 1**	81.21	81.96	86.87	91.47	92.83	93.06	93.36
***Y*** _a*_ **= 2**	80.98	81.58	87.32	91.40	92.68	93.43	93.13
***Y*** _a*_ **= 3**	84.68	85.36	90.19	93.21	94.49	94.87	94.49
***Y*** _a*_ **= 4**	89.81	89.43	92.68	95.62	96.53	96.91	96.75
***Y*** _a*_ **= 5**	92.98	93.21	95.55	97.21	97.74	**97.89**	97.66
***Y*** _a*_ **= 6**	93.36	93.13	95.77	97.13	97.58	97.58	97.43
***Y*** _a*_ **= 7**	81.21	81.96	86.87	91.47	92.83	93.06	93.36

**Table 6:** Average precision rate (APR) and average recall rate (ARR) of different motif co-occurrence histograms.

Descriptor	Performance (%)	Data Set				
		RSSCN7	AID	Outex-13	Outex-14	ETHZ-53
MCM	APR	45.96	22.83	26.85	16.28	29.13
	ARR	1.15	0.68	13.43	8.14	29.13
MCH	APR	**64.10**	**38.04**	**68.60**	**40.91**	**48.38**
	ARR	**1.60**	**1.15**	**34.30**	**20.46**	**48.38**

**Table 7:** Average precision rate (APR) and average recall rate (ARR) of the proposed descriptors.

Descriptor	Performance (%)	Data Set				
		RSSCN7	AID	Outex-13	Outex-14	ETHZ-53
MCH	APR	64.10	38.04	68.60	40.91	48.38
	ARR	1.60	1.15	34.30	20.46	48.38
PUH	APR	72.22	51.04	79.40	76.14	97.81
	ARR	1.81	1.55	39.70	38.07	97.81
HHD	APR	**79.57**	**58.13**	**84.21**	**82.82**	**97.89**
	ARR	**1.99**	**1.76**	**42.10**	**41.41**	**97.89**

**Table 8:** Average precision rate (APR) and average recall rate (ARR) of different methods over RSSCN7, AID, Outex-00013, Outex-00014 and ETHZ-53.

Descriptor	Performance (%)	Data Set				
		RSSCN7	AID	Outex-13	Outex-14	ETHZ-53
mdLBP	APR	73.10	50.81	61.00	48.66	61.43
	ARR	1.83	1.54	30.50	24.33	61.43
maLBP	APR	70.88	47.40	62.54	44.53	55.17
	ARR	1.77	1.43	31.27	22.27	55.17
CDH	APR	73.60	49.50	79.27	74.03	88.53
	ARR	1.84	1.51	39.64	37.02	88.53
MSD	APR	68.44	47.76	70.46	66.32	91.09
	ARR	1.71	1.45	35.23	33.16	91.09
LBP + LNDP	APR	69.46	44.12	70.24	43.86	52.45
	ARR	1.74	1.33	35.12	21.93	52.45
MPEG-CEH	APR	75.39	53.86	78.48	74.41	94.79
	ARR	1.88	1.63	39.24	37.21	94.79
Joint Colorhist	APR	72.82	50.97	77.46	72.97	93.74
	ARR	1.82	1.55	38.73	36.48	93.74
OCLBP	APR	70.70	41.60	77.82	56.13	42.57
	ARR	1.75	1.26	38.91	28.06	42.57
IOCLBP	APR	69.96	44.78	79.58	73.58	45.51
	ARR	1.75	1.35	39.79	36.79	45.51
PUM + MCM	APR	73.94	52.45	81.03	78.13	97.74
	ARR	1.85	1.59	40.51	39.06	97.74
HHD	APR	**79.57**	**58.13**	**84.21**	**82.82**	**97.89**
	ARR	**1.99**	**1.76**	**42.10**	**41.41**	**97.89**

**Table 9:** Feature vector length (D), average retrieval time (s) and memory cost per image (KB) of different descriptors.

Method	Feature Vector Length (D)	Average Retrieval Time (s)	Memory Cost per Image (KB)
mdLBP	2048	3.45	16.00
maLBP	1024	1.74	8.00
CDH	108	0.17	0.84
MSD	78	0.15	0.61
LBP + LNDP	768	1.28	6.00
MPEG-CEH	261	0.45	2.04
Joint Colorhist	512	0.88	4.00
OCLBP	1535	2.55	11.99
IOCLBP	3072	5.24	24.00
PUM + MCM	400/184	0.65	3.13/1.44
HHD	445/229	0.72	3.48/1.79
